# Control Design for Uncertain Higher-Order Networked Nonlinear Systems via an Arbitrary Order Finite-Time Sliding Mode Control Law

**DOI:** 10.3390/s22072748

**Published:** 2022-04-02

**Authors:** Maryam Munir, Qudrat Khan, Safeer Ullah, Tayyaba Maryam Syeda, Abdullah A. Algethami

**Affiliations:** 1 Department of Electrical Engineering, HITEC University, Taxila 47080, Pakistan; maryam_munir96@yahoo.com; 2 Centre for Advanced Studies in Telecommunications (CAST), COMSATS University, Islamabad 45550, Pakistan; qudratullahqau@gmail.com; 3 Department of Electrical and Computer Engineering, COMSATS University, Islamabad 45550, Pakistan; safeer_iiui@yahoo.com (S.U.); s.tayyaba38@gmail.com (T.M.S.); 4 Department of Mechanical Engineering, College of Engineering, Taif University, Taif 11099, Saudi Arabia

**Keywords:** arbitrary order sliding mode, networked system, finite-time systems, nonlinear system

## Abstract

The authors proposed an arbitrary order finite-time sliding mode control (SMC) design for a networked of uncertain higher-order nonlinear systems. A network of n+1 nodes, connected via a directed graph (with fixed topology), is considered. The nodes are considered to be uncertain in nature. A consensus error-based canonical form of the error dynamics is developed and a new arbitrary order distributed control protocol design strategy is proposed, which not only ensures the sliding mode enforcement in finite time but also confirms the finite time error dynamics stability. Rigorous stability analysis, in closed-loop, is presented, and a simulation example is given, which demonstrates the results developed in this work.

## 1. Introduction

In consensus, locally communicating agents reach an agreement which is mostly met via distributed control strategies [1]. These agreements (consensus) finds very impressive applications in formation control [2,3,4], sensor networks [5], smart grid applications [6], and rendezvous control of non-holonomic agents [7,8]. In the context of consensus, cooperative control has been one of the main areas of research, which is subdivided into two main classes called the leaderless control (for instance; [9]), and the leader–follower control [10,11]. In the leader–follower systems, a distributed control strategy is generally designed for the followers to follow the leader, which shares information through a properly defined network topology. Thus far, the leader–follower problems and their solutions via various methodologies, for electro-mechanical (or second-order) systems, is extensively addressed in the existing literature (see, for example; [12,13,14,15,16,17,18,19]). Das and Lewis [14,15] developed distributed laws of adaptive nature for the cooperative tracking of single and double integrator systems in uncertain scenarios. Nonetheless, the requirement of knowing the Laplacian matrix’s non-zero eigenvalue limits its applicability. Cooperative control of higher-order uncertain networked systems was an expansion of [14,15] in Brunovsky form.

The authors, in [11], presented a second-order sliding mode control (SOSMC) technique for the consensus of a network of higher-order nonlinear systems. Their presented results were excellent. However, a distributed law was developed to compensate the bounded uncertainties caused by inputs and states, which raises theoretical concerns. Furthermore, asymptotic convergence does not ensure consensus accuracy. In [20], second-order linear networked systems were designed to compensate matched and mismatched uncertain disturbances. The researchers, in [13], studied a second-order linear network system under an unknown disturbance. Furthermore, second-order SMC based distributed laws were proposed for uncertain second-order linear networked systems in [21] that resulted in finite time error stabilisation. Their presented results were satisfactory; nevertheless, they were confined to linear systems with matching uncertainty. Furthermore, this algorithm demonstrated sensitivity to perturbations during the reaching phase.

An integral SMC law with an extended observer and neural networks was developed to estimate and compensate the uncertain disturbance of matched type, respectively. A distributed control approach based on integral sliding modes (ISM) and subject to fixed topology and the directed graph was devised for uncertain nonlinear networked systems under matched uncertainties [22]. This technique alleviates the reaching phase, resulting in increased robustness. It was, nonetheless, confined to electromechanical systems. In terms of applications, Ref. [23] proposed adaptive formation control algorithms for a class of non-holonomic mobile robots. These approaches mainly focused on the stability of a network of linear and nonlinear second-order agents even in bounded uncertainties. However, their performance can be affected due to the existence of all system dynamics in the sliding manifold.

To maintain the convergence of finite-time consensus mismatches at zero, a distributed control system based on a terminal sliding mode control (TSMC) technique was devised. However, the existence of the singularity in the surface may reduce its significance [24,25]. In [12], an uncertain network of first-order Multi Input Multi-Output (MIMO) systems was focused where neural networks (NNs) were utilized for the uncertain dynamic estimation. In order to alleviate the approximation error, a robustness signal was also used. Nevertheless, it was ultimately bounded. The control researcher, in [26], investigated uncertain MIMO second-order networked systems with a fixed topology and undirected graph and developed a distributed TSMC, based on Chebyshev Neural Networks (CNNs), to compensate the external disturbances and uncertain dynamics. An approach based on NNs was designed to estimate the uncertain input channels and drift terms and compensate the uncertainties. Nevertheless, this strategy was influential in the asymptotic stability of tracking error dynamics to the limited neighborhood of the origin. Ref. [27] investigated networked MIMO higher-order systems for synchronization applications. While applying NNs, these MIMO agents were controlled through the unknown non-singular control gains. The limits of the error dynamics may not have been easily decreased by modifying the controller gains. The control gains must be properly selected to guarantee the asymptotic convergence. Ref. [28]’s methodology was enhanced in [29] by including a neuro-adaptive sliding mode strategy. However, this led to several limitations, such as the fact that the boundedness of the approximated NNs’ weights cannot always be ensured using the proposed tuning laws. Additionally, ensuring the boundedness of the control input is quite challenging.

At this stage, it was realized to develop a terminal sliding mode like a strategy that must confirm finite time error dynamics convergence and show robustness to cross-coupling of the agents and matched disturbances from the very beginning. Therefore, this paper studies the cooperative tracking control of higher-order nonlinear systems subject to uncertainties like parametric variations and matched bounded disturbances. The distributed control laws are developed on novel sliding surfaces of the error dynamics. The designed sliding manifold, which involves some discontinuous terms of the errors, seems analogous to the proportional-integral type, which helps in the elimination of the critical reaching phase. Consequently, robustness is guaranteed from the very start. Having established sliding modes, the error dynamics seem analogous to terminal attractor like in [30] which exhibits finite convergence. Thus, all the error dynamics converge in finite time, which results in high precision. In addition, our proposed work solves the theoretical shortcoming of [11] and the uncertain terms are now depending on the states information of the agents. The rest of the paper is organised as follows: Section 2 is about the problem formulation and mathematical preliminaries. In Section 3 and Section 4, the detailed controller design procedure and the stability analysis are discussed, respectively. The illustrative example is mentioned in Section 5. Finally, Section 6 concludes the article.

## 2. Problem Formulation

### Definitions

In this study, a network of n+1 nodes is considered which share information via a directed graph (with fixed topology). These networked nodes include one leader and *n* followers. The followers are assumed under the action of uncertainties. The following state space equations represent the dynamical model of an *i*th follower:

(1) $$\begin{array}{cc} {\dot{x}}_{ij}= & x_{ij+1}\;\\ {\dot{x}}_{in}= & f_{i}\left(x_{i}\right)+g_{i}\left(x_{i}\right)u_{i}+\Delta_{i}\left(x_{i},t\right) \end{array}$$

where i=1,2,…,n, j=1,2,…,n−1, $x_{i}={\left[x_{i1},x_{i2},\ldots ,x_{in}\right]}^{T}\in {ℜ}^{n}$ is the measurable state vector, $\Delta_{i}\left(x_{i},t\right)$ is the uncertainty, $u_{i}$ represents the control input which is to be applied to the system, and $f_{i}$ and $g_{i}$ are the system distribution and drift functions, respectively. For the sake of the detailed description, the following assumptions are made:

**Assumption** **A1.** *It is assumed that $g_{i}\left(x_{i}\right)$*∀*$x_{i}\in {ℜ}^{n}$ is non singular, which will guarantee the controllability of each network agent.*

**Assumption** **A2.** *The uncertainty $\Delta_{i}\left(x_{i},t\right)$ is assumed to be norm bounded i.e.,*

(2) $$\left|\right|\Delta_{i}\left(x_{i},t\right)\left|\right|\leq C_{i}$$

*where i=1,2,…,n, ||.||, and $C_{i}$ represents Euclidean norm and a positive constant, respectively.*

The leader is governed by the following state space model:

(3) $$\begin{array}{cc} {\dot{x}}_{0r}= & x_{0r+1},\\ {\dot{x}}_{0n}= & f_{0}\left(t,x_{0}\right) \end{array}$$

where r=1,2…,n−1, $x_{0}={\left[x_{01},x_{02},\ldots ,x_{0n}\right]}^{T}\in {ℜ}^{n}$ is state vector of the leader and $f_{0}\left(t,x_{0}\right)$ is the continuous bounded function, which derive the leader. Suppose that the origin is an equilibrium for $f_{0}\left(t,x_{0}\right)$ i.e., $f_{0}\left(t,0\right)=0$ and the nonlinear function $f_{0}\left(t,x_{0}\right)$ (leader driving force) is considered to be bounded and smooth. The vector set $V=\{V_{0},V_{1},\ldots ,V_{n}\}$ represents the relationship between the leader and the follower nodes while G={V,E} is the related directed graph in which node *i* can transfer data with node *j*, but node *j* cannot send back the information to node *i*. On the other hand, in an undirected graph, both way communication takes place. The mathematical expression for the adjacency matrix is given as follows:

$$A_{i}=\left[\begin{array}{cccc} 0 & 0 & \ldots & 0\\ a_{10} & a_{11} & \ldots & a_{1n}\\ a_{20} & a_{21} & \ldots & a_{2n}\\ \vdots & \ldots & \ddots & \vdots \\ a_{n0} & a_{n1} & \ldots & a_{nn} \end{array}\right]$$

Subgraph $\bar{G}=\left\{\bar{V},\bar{E}\right\}$ can be obtained by dropping the first row and first column of the above adjacency matrix; thus, one has

$$\bar{A_{i}}=\left[\begin{array}{cccc} a_{11} & a_{12} & \ldots & a_{1n}\\ a_{21} & a_{22} & \ldots & a_{2n}\\ \ldots & \ldots & \ddots & \vdots \\ a_{n1} & a_{n2} & \ldots & a_{nn} \end{array}\right]$$

The Laplacian matrix for the followers topology is defined to be $\bar{L}=\bar{D}-\bar{A}\in {ℜ}^{n\times n}$, where $D=diag[\bar{d_{1}},\bar{d_{2}},\ldots ,\bar{d_{n}}]$ with $\bar{d_{i}}=\sum_{j=1}^{n}a_{ij}$. In addition, note that $a_{ij}=0$ if $(V_{j},V_{i})\notin E$ and $a_{ij}=0$ otherwise. The matrix $\bar{B}=diag\left[b_{1},b_{2},\ldots ,b_{n}\right]$ shows the connection between the followers and the leader with $b_{i}=0$ if the follower is not connected to the leader and $b_{i}=1$ in case of the connection to the leader. A is time-invariant throughout the paper. Since we are considering a directed graph, matrix A is not necessarily to be symmetric. In contrast, in the case of the undirected graph, the symmetry is necessary for A. $\bar{L}+\bar{B}$ must be non-singular for the distributed control of all the networked agents. Similarly, $\bar{D}$ remains non-singular.

The main objective of the current work is that the follower states must have consensus with the leader states (in other words, the followers must follow the leader). In order to complete the task, the consensus error between the leader and the *i*th follower must be forced to zero. Therefore, the consensus error is defined as follows:

(4) $$e_{ik}=\sum_{j=1,j\neq i}^{n}a_{ij}\left(x_{ik}-x_{jk}\right)+b_{i}\left(x_{ik}-x_{0k}\right)$$

where k=1,2,..,n. Based on the consensus error Equation (4), the consensus error dynamics can be expressed as follows:

$${\dot{e}}_{i1}=e_{i2}$$

$${\dot{e}}_{i2}=e_{i3}\;\vdots$$

(5) $$\begin{array}{cc} {\dot{e}}_{in}= & \left(\sum_{j=1,j\neq i}^{n}a_{ij}+b_{i}\right)\left(f_{i}\left(x\right)+g_{i}\left(x\right)u_{i}\right)-\sum_{j=1,j\neq i}^{n}a_{ij}\left(f_{j}\left(x\right)+g_{j}\left(x\right)u_{j}\right)\\ \; & -b_{i}f_{0}\left(x,t\right)+h_{i}\left(x,t\right) \end{array}$$

with

$$\begin{array}{c} h_{i}\left(x,t\right)=\left(\sum_{j=1,j\neq i}^{n}a_{ij}+b_{i}\right)\Delta_{i}\left(x,t\right)-\sum_{j=1,j\neq i}^{n}a_{ij}\Delta_{j}\left(x,t\right) \end{array}$$

representing the uncertainty terms in lumped form. In this equation, it is clearly shown that the uncertainties depends only on the system states.

**Remark** **1.** *The compact form of (5) can also be written in the following form:*

(6) $$\begin{array}{cc} {\dot{\Sigma }}_{1}= & \Sigma_{2}\\ {\dot{\Sigma }}_{2}= & \Sigma_{3}\\ \vdots \\ {\dot{\Sigma }}_{n}= & \left(\bar{L}+\bar{B}\right)\left(f\left(x\right)+g\left(x\right)u+\Delta \left(x,t\right)-\bar{1}f_{0}\left(t,x\right)\right) \end{array}$$

*where*

$$\Sigma_{1}=\left[e_{11},e_{21},e_{31},\ldots ,e_{n1}\right],\;\Sigma_{2}=\left[e_{12},e_{22},e_{32},\ldots ,e_{n2}\right],\;\vdots \;\Sigma_{n}=\left[e_{1n},e_{2n},e_{3n},\ldots ,e_{nn}\right],$$

*and $f\left(x\right)={\left[f_{1}\left(x_{1}\right),f_{2}\left(x_{2}\right),\ldots ,f_{n}\left(x_{n}\right)\right]}^{T}$, $g\left(x\right)=diag[g_{1}\left(x_{1}\right),g_{2}\left(x_{2}\right),\ldots ,g_{n}\left(x_{n}\right)]$, $\bar{1}={\left[11\ldots 1\right]}^{T}$, $u={\left[u_{1},u_{2},\ldots ,u_{n}\right]}^{T}$ and $\Delta \left(t,x\right)={\left[\Delta_{1}\left(t,x_{1}\right),\Delta_{2}\left(t,x_{2}\right),\ldots ,\Delta_{n}\left(t,x_{n}\right)\right]}^{T}$.*

Now, the problem at hand becomes an error regulation problem which will, in other words, provide a consensus among the leader and *n* followers. The task can be accomplished by a robust nonlinear sliding mode strategy which will nullify the effects of uncertain terms and will ensure finite time error dynamics convergence. In the following section, a novel finite-time sliding mode strategy is presented.

## 3. Control Problem Design

The main task here is to drive the error dynamics (5) states to the equilibrium in the presence of disturbances. To achieve this goal, a novel sliding surface based sliding mode control protocol is presented. The proposed sliding surface helps in the finite time convergence of the consensus error dynamics (5) to equilibrium and also establishes finite time sliding mode. The newly proposed sliding surface, for follower *i*, can be defined as follows:

(7) $$\begin{array}{cc} s_{i} & =\left(e_{in}+\sum_{j=1}^{n-1}a_{ij}e_{ij}\right)+\int_{0}^{t}\sum_{j=1}^{n}\left(b_{ij}{\left|e_{ij}\right|}^{\alpha_{ij}}sign\left(e_{ij}\right)\right.+\left.c_{ij}{\left|e_{ij}\right|}^{\beta_{ij}}sign\left(e_{ij}\right)\right)d\tau \end{array}$$

In expanded form, this surface can be defined as follows:

(8) $$\begin{array}{cc} s_{i} & =e_{in}+a_{i1}e_{i1}+a_{i2}e_{i2}+\ldots +a_{i(n-1)}e_{i(n-1)}\\ \; & +\int_{0}^{t}\left(b_{i1}|e_{i1}{|}^{\alpha_{i1}}sign\left(e_{i1}\right)+\ldots +b_{in}{\left|e_{in}\right|}^{\alpha_{in}}sign\left(e_{in}\right)\right.\\ \; & \left.+c_{i1}|e_{i1}{|}^{\beta_{i1}}sign\left(e_{i1}\right)+\ldots +c_{in}{\left|e_{in}\right|}^{\beta_{in}}sign\left(e_{in}\right)\right)d\tau \end{array}$$

**Remark** **2.** *In the above Equation (8), the terms $\alpha_{i}$ and $\beta_{i}$ are chosen as follows [31]:*

$$\alpha_{i-1}=\frac{\alpha_{i}\alpha_{i+1}}{2\alpha_{i+1}-\alpha_{i}},\beta_{i}=\frac{\beta_{i}\beta_{i+1}}{2\beta_{i+1}-\beta_{i}}$$

*where $\alpha_{n+1}=1$, $\alpha_{n}=\alpha$ and $\beta_{n+1}=1$, $\beta_{n}=\beta$, α, β∈ℜ. In addition, $\alpha_{i}\in \left(0,1\right)$ and $\beta_{i}\in \left(1,1+\in \right)$ where ∈ > 0.*

By taking the derivative of the above equation, one may obtain the following expression:

(9) $$\begin{array}{cc} \dot{s_{i}} & ={\dot{e}}_{in}+a_{i1}{\dot{e}}_{i1}+a_{i2}{\dot{e}}_{i2}+\ldots +a_{i(n-1)}{\dot{e}}_{i(n-1)}\\ \; & +\left(b_{i1}|e_{i1}{|}^{\alpha_{i1}}sign\left(e_{i1}\right)+\ldots +b_{in}{\left|e_{in}\right|}^{\alpha_{in}}sign\left(e_{in}\right)\right.\\ \; & \left.+c_{i1}|e_{i1}{|}^{\beta_{i1}}sign\left(e_{i1}\right)+\ldots +c_{in}{\left|e_{in}\right|}^{\beta_{in}}sign\left(e_{in}\right)\right) \end{array}$$

Substituting the values from (5) in (9), it becomes as follows:

(10) $$\begin{array}{cc} \dot{s_{i}} & =\left(\sum_{j=1,j\neq i}^{n}a_{ij}+b_{i}\right)\left(f_{i}\left(x\right)+g_{i}\left(x\right)u_{i}\right)\\ \; & -\sum_{j=1,j\neq i}^{n}a_{ij}\left(f_{j}\left(x\right)+g_{j}\left(x\right)u_{j}\right)-b_{i}f_{0}\left(x,t\right)+h_{i}\left(x,t\right)\\ \; & +a_{i1}e_{i2}+a_{i2}e_{i3}+\ldots +a_{i(n-1)}e_{in}\\ \; & +b_{i1}|e_{i1}{|}^{\alpha_{i1}}sign\left(e_{i1}\right)+\ldots +b_{in}{\left|e_{in}\right|}^{\alpha_{in}}sign\left(e_{in}\right)\\ \; & +c_{i1}|e_{i1}{|}^{\beta_{i1}}sign\left(e_{i1}\right)+\ldots +c_{in}{\left|e_{in}\right|}^{\beta_{in}}sign\left(e_{in}\right) \end{array}$$

Now, our objective is to calculate the equivalent control law [29], to ensure the Filippove sense solutions [32] in sliding modes. Posing ${\dot{s}}_{i}=0$ and calculating for $u_{i}$, while, assuming $h_{i}\left(x,t\right)=0$, one may obtain

(11) $$\begin{array}{cc} u_{i(eq)} & ={\left(\left(\sum_{j=1,j\neq i}^{n}a_{ij}+b_{i}\right)g_{i}\right)}^{-1}\times \left(-\left(\sum_{j=1,j\neq i}^{n}a_{ij}+b_{i}\right)f_{i}\right.\\ \; & +\sum_{j=1,j\neq i}^{n}a_{ij}\left(f_{j}+g_{j}u_{j}\right)+b_{i}f_{0}\left(x,t\right)\\ \; & -a_{i1}e_{i2}-a_{i2}e_{i3}-\ldots -a_{i(n-1)}e_{in}\\ \; & -b_{i1}|e_{i1}{|}^{\alpha_{i1}}sign\left(e_{i1}\right)-\ldots -b_{in}{\left|e_{in}\right|}^{\alpha_{in}}sign\left(e_{in}\right)\\ \; & \left.-c_{i1}|e_{i1}{|}^{\beta_{i1}}sign\left(e_{i1}\right)-\ldots -c_{in}{\left|e_{in}\right|}^{\beta_{in}}sign\left(e_{in}\right)\right) \end{array}$$

This control component governs the system trajectories exactly on the sliding surface $s_{i}=0$ [1]. To ensure the robustness against uncertainties of a matched kind, the overall control law is considered as an algebraic sum of the aforementioned equivalent control component and a discontinuous control component i.e.,

(12) $$u_{i}=u_{i(eq)}+u_{i(dis)}$$

where

(13) $$u_{i(dis)}=-K_{i}sign\left(s_{i}\right)$$

with $K_{i}$ being the switching gain. Thus, the final distributed control protocol can be obtained by putting (11) and (13) in (12). The control law defined in (12) ensures the convergence of system states to zero in finite time. The following stability analysis presents the detailed presentation of sliding mode enforcement and the finite-time convergence of the system states.

## 4. Stability Analysis

Now, at this stage, it is necessary to present the stability of the proposed control protocol in a close loop under the effect of the uncertainty. Therefore, the following theorem is stated.

**Theorem** **1.** *The finite sliding mode can be enforced along the nonlinear sliding surface (8) by the control protocol (12). If the switching gain is chosen as follows*

$$K_{i}\geq \left|h_{i}\left(x,t\right)\right|+\eta ,$$

*the trajectories of (5) also converge in finite time to the equilibrium.*

**Proof.**  A Lyapunov function of the following form is considered to prove the theorem:

(14) $$v_{i}\left(s_{i}\right)=\frac{1}{2}s_{i}^{2}$$

The time derivative of (14) along (10) becomes

$${\dot{v}}_{i}\left(s_{i}\right)=s_{i}{\dot{s}}_{i}$$

(15) $$\begin{array}{cc} {\dot{v}}_{i}\left(s_{i}\right)= & s_{i}\left(\left(\sum_{j=1,j\neq i}^{n}a_{ij}+b_{i}\right)\left(f_{i}\left(x\right)+g_{i}\left(x\right)u_{i}\right)\right.-\sum_{j=1,j\neq i}^{n}a_{ij}\left(f_{i}\left(x\right)+g_{i}\left(x\right)u_{i}\right)\\ \; & -b_{i}f_{0}\left(x,t\right)+h_{i}\left(x,t\right)+a_{i1}e_{i2}+a_{i2}e_{i3}+\ldots +a_{i(n-1)}e_{in}\\ \; & +b_{i1}|e_{i1}{|}^{\alpha_{i1}}sign\left(e_{i1}\right)+\ldots +b_{in}{\left|e_{in}\right|}^{\alpha_{in}}sign\left(e_{in}\right)\\ \; & +\left.c_{i1}|e_{i1}{|}^{\beta_{i1}}sign\left(e_{i1}\right)+\ldots +c_{in}{\left|e_{in}\right|}^{\beta_{in}}sign\left(e_{in}\right)\right) \end{array}$$

Incorporating (12) in (15) (with components in (11) and (13)), it reduces to

$${\dot{v}}_{i}\left(s_{i}\right)=s_{i}\left(h_{i}\left(x,t\right)-K_{i}sign\left(s_{i}\right)\right)$$

(16) $${\dot{v}}_{i}\left(s_{i}\right)=s_{i}h_{i}\left(x,t\right)-s_{i}K_{i}sign\left(s_{i}\right)$$

Using the identity $s_{i}sign\left(s_{i}\right)=\left|s_{i}\right|$, (16) becomes

$${\dot{v}}_{i}\left(s_{i}\right)=s_{i}h_{i}\left(x,t\right)-K_{i}\left|s_{i}\right|$$

$${\dot{v}}_{i}\left(s_{i}\right)\leq |s_{i}\left|\right|h_{i}\left(x,t\right)|-K_{i}\left|s_{i}\right|$$

(17) $${\dot{v}}_{i}\left(s_{i}\right)\leq -|s_{i}|(K_{i}-|h_{i}\left(x,t\right)\left|\right)$$

The sliding mode along (8) can be ensured, if one chooses

(18) $$K_{i}-\left|h_{i}\left(x,t\right)\right|\geq \eta_{i}>0$$

$$K_{i}\geq \left|h_{i}\left(x,t\right)\right|+\eta_{i}$$

where $\eta_{i}$ refers to small positive constants. Thus, using (18), (17) becomes

$${\dot{v}}_{i}=-\eta_{i}\left|s_{i}\right|$$

(19) $${\dot{v}}_{i}\leq -\sqrt{2}\eta_{i}{v_{i}}^{1/2}$$

The time $t_{s}$ taken for the trajectory of the proposed system to reach the sliding surface can be found by integrating (19) as

$$t_{s}\leq \frac{1}{2{\bar{\eta }}_{i}}\ln\left({\bar{\eta }}_{i}v^{\frac{1}{2}}s_{i}\left(0\right)\right):\;\;\;\;\mathrm{where}\;\;{\bar{\eta }}_{i}=\sqrt{2}\eta_{i}$$

This equation certifies the finite time convergence of sliding mode along (8) [33]. The establishment of sliding mode along (8) means that the system trajectories now evolve on the manifold $s_{i}=0$. Thus, one may have

(20) $$\begin{array}{cc} \; & e_{in}+a_{i1}e_{i1}+a_{i2}e_{i2}+\ldots +a_{i(n-1)}e_{i(n-1)}\\ \; & +\int_{0}^{t}\left(b_{i1}|e_{i1}{|}^{\alpha_{i1}}sign\left(e_{i1}\right)+\ldots +b_{in}{\left|e_{in}\right|}^{\alpha_{in}}sign\left(e_{in}\right)\right.\\ \; & \left.+c_{i1}|e_{i1}{|}^{\beta_{i1}}sign\left(e_{i1}\right)+\ldots +c_{in}{\left|e_{in}\right|}^{\beta_{in}}sign\left(e_{in}\right)\right)d\tau =0 \end{array}$$

Equation (20) can also be written as

(21) $$\begin{array}{cc} \; & {\dot{e}}_{in}+b_{in}|e_{in}{|}^{\alpha_{in}}sign\left(e_{in}\right)+c_{in}{\left|e_{in}\right|}^{\beta_{in}}sign\left(e_{in}\right)\\ \; & +(a_{i(n-1)}{\dot{e}}_{i(n-1)}+b_{i(n-1)}|e_{i(n-1)}{|}^{\alpha_{i(n-1)}}sign\left(e_{i(n-1)}\right)\\ \; & +c_{i(n-1)}{\left|e_{i(n-1)}\right|}^{\beta_{i(n-1)}}sign\left(e_{i(n-1)}\right)+\ldots +a_{i1}{\dot{e}}_{i1}\\ \; & +b_{i1}|e_{i1}{|}^{\alpha_{i1}}sign\left(e_{i1}\right)+c_{i1}{\left|e_{i1}\right|}^{\beta_{i1}}sign\left(e_{i1}\right)=0 \end{array}$$

Equation (21) holds only if

$${\dot{e}}_{in}+b_{in}|e_{in}{|}^{\alpha_{in}}sign\left(e_{in}\right)+c_{in}{\left|e_{in}\right|}^{\beta_{in}}sign\left(e_{in}\right)=0$$

$$\begin{array}{cc} \; & {\dot{e}}_{i(n-1)}+\frac{b_{i(n-1)}}{a_{i(n-1)}}{\left|e_{i(n-1)}\right|}^{\alpha_{i(n-1)}}sign\left(e_{i(n-1)}\right)\\ \; & +\frac{c_{i(n-1)}}{a_{i(n-1)}}{\left|e_{i(n)}\right|}^{\beta_{i(n-1)}}sign\left(e_{i(n-1)}\right)=0 \end{array}$$

⋮

(22) $${\dot{e}}_{i1}+\frac{b_{i1}}{a_{i1}}|e_{i1}{|}^{\alpha_{i1}}sign\left(e_{i1}\right)+\frac{c_{i1}}{a_{i1}}{\left|e_{i1}\right|}^{\beta_{i1}}sign\left(e_{i1}\right)=0$$

These equations in (22) are finite time stable terminal attractors [30], which confirm that $e_{ij}\to 0$ in finite time and stays at zero for all subsequent times. This proves the theorem. □

## 5. Illustrative Example

**Figure 1:**
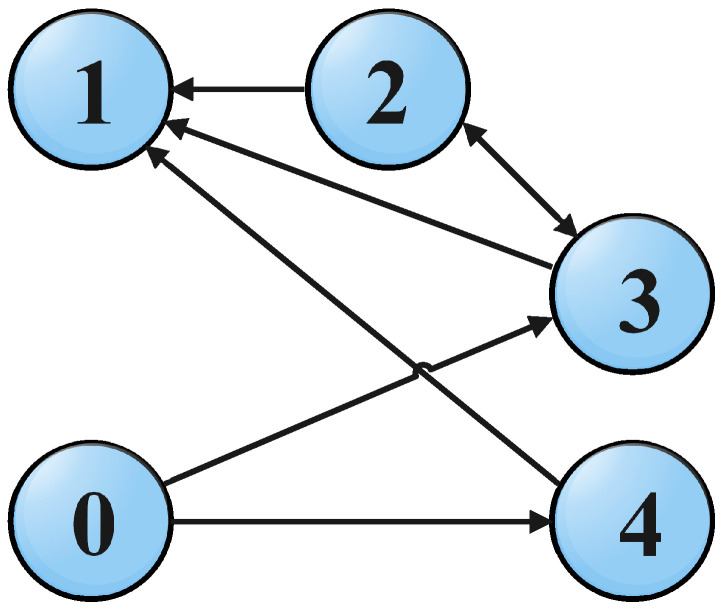
Topology of the system network of one leader and four followers.

This design strategy presented above is validated in this section via the simulation study of a numerical example. The example is conducted according to the topology shown in Figure 1, which consists of one leader and four followers. The leader and the followers, considered here, are governed by third-order uncertain systems. In addition, the agents are operated under uncertainties of the matched kind. The descriptions of the considered systems are presented in the following study.

### 5.1. Systems Description

The dynamics of the leader are

(23) $$\begin{array}{cc} {\dot{x}}_{01}= & x_{02}\\ {\dot{x}}_{02}= & x_{03}\\ {\dot{x}}_{03}= & -x_{02}-2x_{03}+1+3\sin\left(2t\right)+2\cos\left(2t\right) \end{array}$$

The governing equations of the followers are written as follows:

(24) $$\begin{array}{cc} {\dot{x}}_{13} & =x_{12}\sin\left(x_{11}\right)+\cos^{2}\left(x_{13}\right)+\left(0.1+x_{12}^{2}\right)u_{1}+\xi_{1}\\ {\dot{x}}_{23} & =-x_{21}x_{22}+0.01x_{21}-0.01x_{21}^{2}+\left(1+\sin^{2}\left(x_{21}\right)\right)u_{2}+\xi_{2}\\ {\dot{x}}_{33} & =x_{32}+\sin\left(x_{33}\right)+\left(1+\cos^{2}\left(x_{32}\right)\right)u_{3}+\xi_{3}\\ {\dot{x}}_{43} & =-3{\left(x_{41}+x_{42}-1\right)}^{2}\left(x_{41}+x_{42}+x_{43}-1\right)-x_{42}\\ \; & -x_{43}+0.5\sin\left(2t\right)+\cos\left(2t\right)+\left(1+0.5x_{42}^{2}\right)u_{4}+\xi_{4} \end{array}$$

where the term $\xi_{i}$ represents the matched uncertainties in the follower dynamics. That is, $\xi_{1}$ is matched uncertainty in follower 1 and so on.

Since the graph is directed, so the Laplacian and adjacency matrices are written as follows:

$$A=\left[\begin{array}{ccccc} 0 & 0 & 0 & 0 & 0\\ 0 & 0 & 1 & 1 & 1\\ 0 & 0 & 0 & 1 & 0\\ 1 & 0 & 1 & 0 & 0\\ 1 & 0 & 0 & 0 & 0 \end{array}\right]\;\bar{L}=\left[\begin{array}{cccc} 3 & -1 & -1 & -1\\ 0 & 1 & -1 & 0\\ 0 & -1 & 1 & 0\\ 0 & 0 & 0 & 0 \end{array}\right]$$

and

$$\bar{B}=diag\left[0,0,1,1\right]$$

The main task here is that the followers should follow the leader trajectory. For achieving this task, one needs to design a controller by following the steps mentioned in the previous section.

### 5.2. Controller Design

Since four followers and one leader are considered, the consensus errors, which will be steered to zero, are therefore defined as follows:

$$e_{ij}=\sum_{j=1}^{4}a_{ij}\left(x_{i1}-x_{j1}\right)+b_{i}\left(x_{i1}-x_{01}\right);\;\;\;i=1,2,3,4$$

The related sliding manifolds are defined as follows:

(25) $$\begin{array}{cc} s_{i} & =e_{i4}+a_{i3}e_{i3}+a_{i2}e_{i2}+a_{i1}e_{i1}+\\ \; & \int_{0}^{t}b_{i1}|e_{i1}{|}^{\alpha_{i1}}sign\left(e_{i1}\right)+\ldots +b_{i4}{\left|e_{i4}\right|}^{\alpha_{i4}}sign\left(e_{i4}\right)\\ \; & +c_{i1}|e_{i1}{|}^{\beta_{i1}}sign\left(e_{i1}\right)+\ldots +c_{i4}{\left|e_{i4}\right|}^{\beta_{i4}}sign\left(e_{i4}\right)d\tau \end{array}$$

where i=1,2,3,4.

The expression for controllers are given below

(26) $$\begin{array}{cc} u_{1}= & {\left(\left(\sum_{j=1,j\neq i}^{4}a_{1j}+b_{1}\right)g_{i}\right)}^{-1}\times \left(-\left(\sum_{j=1,j\neq i}^{4}a_{1j}+b_{1}\right)f_{1}+\sum_{j=1,j\neq i}^{4}a_{1j}\left(f_{1}+g_{1}u_{1}\right)\right.\\ \; & +b_{1}f_{0}\left(x,t\right)-a_{11}e_{12}-a_{12}e_{13}-\ldots -a_{13}e_{14}\\ \; & -b_{11}|e_{11}{|}^{\alpha_{11}}sign\left(e_{11}\right)-\ldots -b_{14}{\left|e_{14}\right|}^{\alpha_{14}}sign\left(e_{14}\right)\\ \; & \left.-c_{11}|e_{11}{|}^{\beta_{11}}sign\left(e_{11}\right)-\ldots -c_{14}{\left|e_{14}\right|}^{\beta_{14}}sign\left(e_{1n}\right)\right)\\ \; & -u_{1(dis)} \end{array}$$

(27) $$\begin{array}{cc} u_{2}= & {\left(\left(\sum_{j=1,j\neq i}^{4}a_{2j}+b_{2}\right)g_{2}\right)}^{-1}\times \left(-\left(\sum_{j=1,j\neq i}^{4}a_{2j}+b_{2}\right)f_{2}+\right.\sum_{j=1,j\neq i}^{4}a_{2j}\left(f_{2}+g_{2}u_{2}\right)\\ \; & +b_{2}f_{0}\left(x,t\right)-a_{21}e_{22}-a_{22}e_{23}-\ldots -a_{23}e_{24}-\\ \; & b_{21}|e_{21}{|}^{\alpha_{21}}sign\left(e_{21}\right)-\ldots -b_{24}{\left|e_{24}\right|}^{\alpha_{24}}sign\left(e_{24}\right)-\\ \; & c_{21}|e_{21}{|}^{\beta_{21}}sign\left(e_{21}\right)-\ldots -c_{24}{\left|e_{24}\right|}^{\beta_{24}}sign\left(e_{24}\right))\\ \; & -u_{2(dis)} \end{array}$$

(28) $$\begin{array}{cc} u_{3}= & {\left(\left(\sum_{j=1,j\neq i}^{4}a_{3j}+b_{3}\right)g_{3}\right)}^{-1}\times \left(-\left(\sum_{j=1,j\neq i}^{4}a_{3j}+b_{3}\right)f_{3}\right.+\sum_{j=1,j\neq i}^{4}a_{3j}\left(f_{3}+g_{3}u_{3}\right)\\ \; & +b_{3}f_{0}\left(x,t\right)-a_{31}e_{32}-a_{32}e_{33}-\ldots -a_{33}e_{34}-\\ \; & b_{31}|e_{31}{|}^{\alpha_{31}}sign\left(e_{31}\right)-\ldots -b_{34}{\left|e_{34}\right|}^{\alpha_{34}}sign\left(e_{34}\right)-\\ \; & c_{31}|e_{31}{|}^{\beta_{31}}sign\left(e_{31}\right)-\ldots -c_{34}{\left|e_{34}\right|}^{\beta_{34}}sign\left(e_{34}\right))\\ \; & -u_{3(dis)} \end{array}$$

(29) $$\begin{array}{cc} u_{4} & ={\left(\left(\sum_{j=1,j\neq i}^{4}a_{4j}+b_{4}\right)g_{4}\right)}^{-1}\times \left(-\left(\sum_{j=1,j\neq i}^{4}a_{4j}+b_{4}\right)f_{4}\right.+\sum_{j=1,j\neq i}^{4}a_{4j}\left(f_{4}+g_{4}u_{4}\right)\\ \; & +b_{4}f_{0}\left(x,t\right)-a_{41}e_{42}-a_{42}e_{43}-\ldots -a_{43}e_{44}-\\ \; & b_{41}|e_{41}{|}^{\alpha_{41}}sign\left(e_{41}\right)-\ldots -b_{44}{\left|e_{44}\right|}^{\alpha_{44}}sign\left(e_{44}\right)-\\ \; & c_{41}|e_{41}{|}^{\beta_{41}}sign\left(e_{41}\right)-\ldots -c_{44}{\left|e_{44}\right|}^{\beta_{44}}sign\left(e_{44}\right))\\ \; & -u_{4(dis)} \end{array}$$

These distributed control algorithms are used in the closed-loop, and the consensus with the leader trajectories is met, which will be discussed below.

### 5.3. The Simulation Results’ Discussion

The network of the four followers agents and one leader, which we are sharing information through the network topology shown in Figure 1 are simulated under the action of the distributed control protocols designed above. The leader and followers were initialized from different initial conditions, and the controller’s gains were chosen according to the values reported in Table 1. The simulations are performed in the MATLAB environment while using an S-function. The numerical solver is used with a fixed step Euler Method with a step size of 0.01 s.

**Figure 2:**
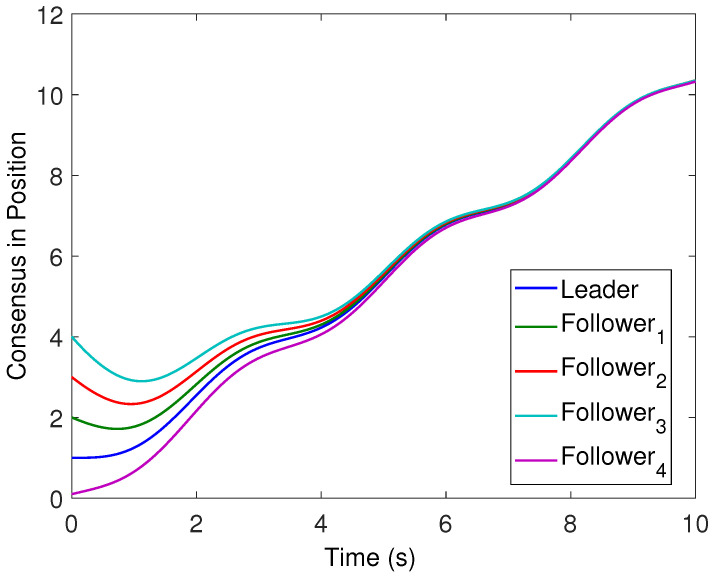
Position consensus of the four followers with leader position trajectory.

**Figure 3:**
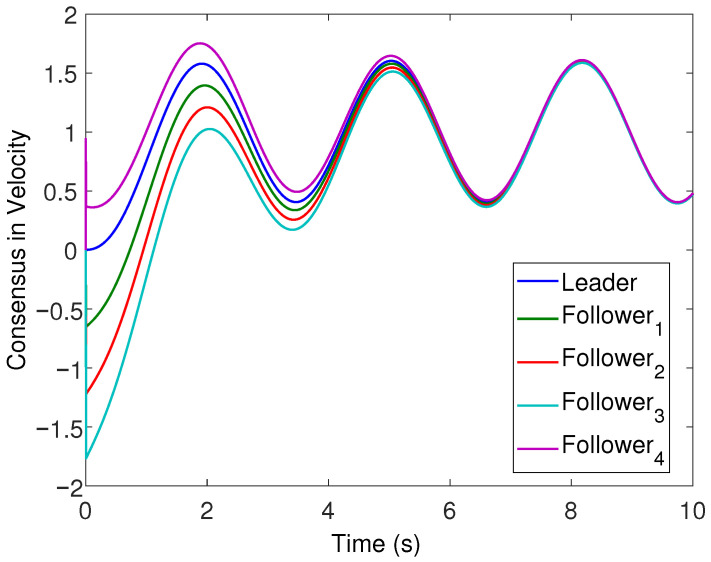
Velocity consensus of the four followers with leader velocity trajectory.

**Figure 4:**
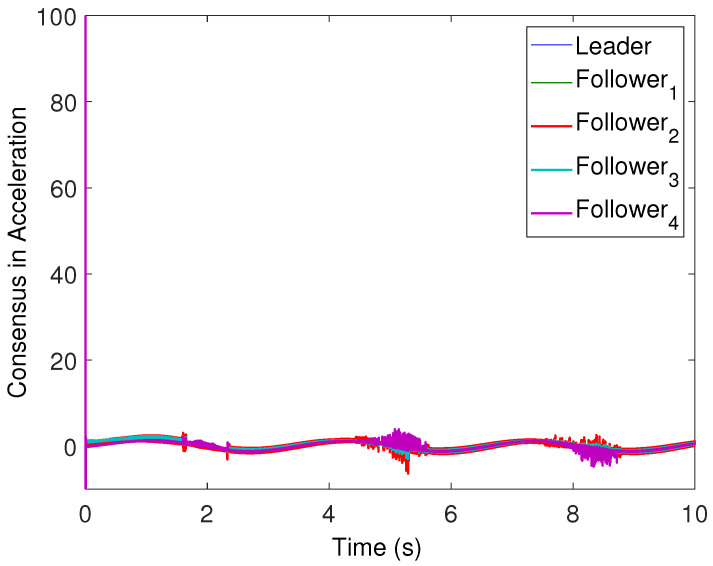
Acceleration consensus of the four followers with leader acceleration trajectory.

**Figure 5:**
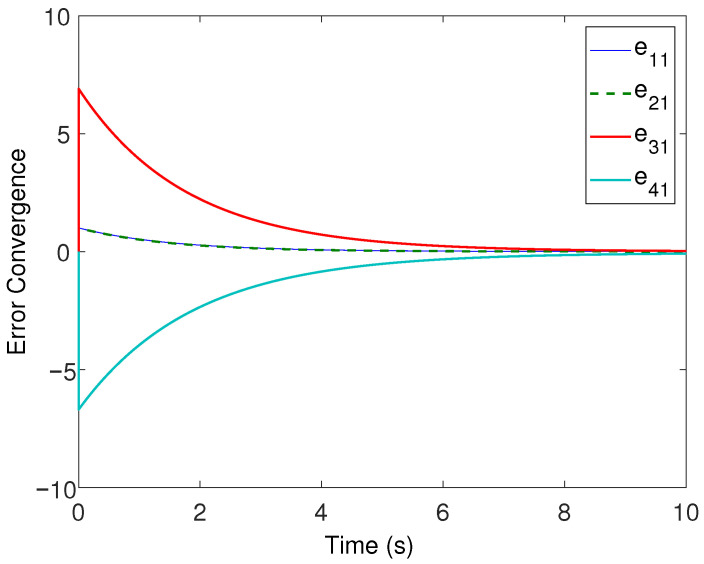
Position errors’ convergence.

**Figure 6:**
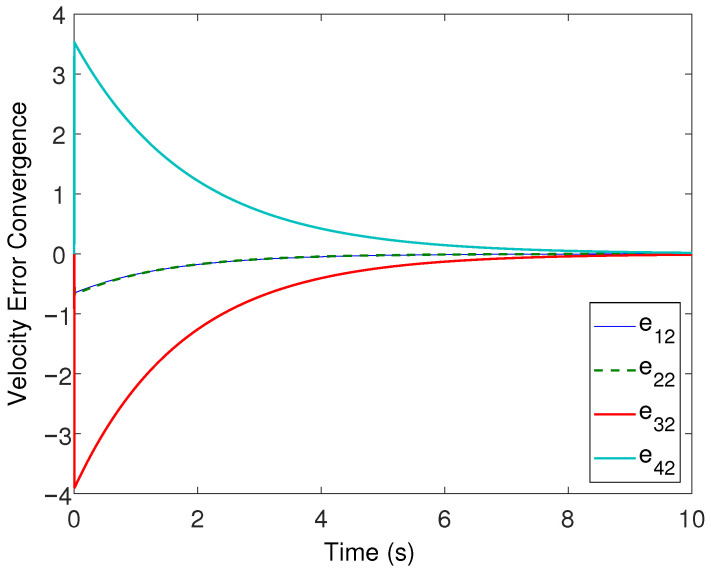
Velocity errors’ convergence.

**Figure 7:**
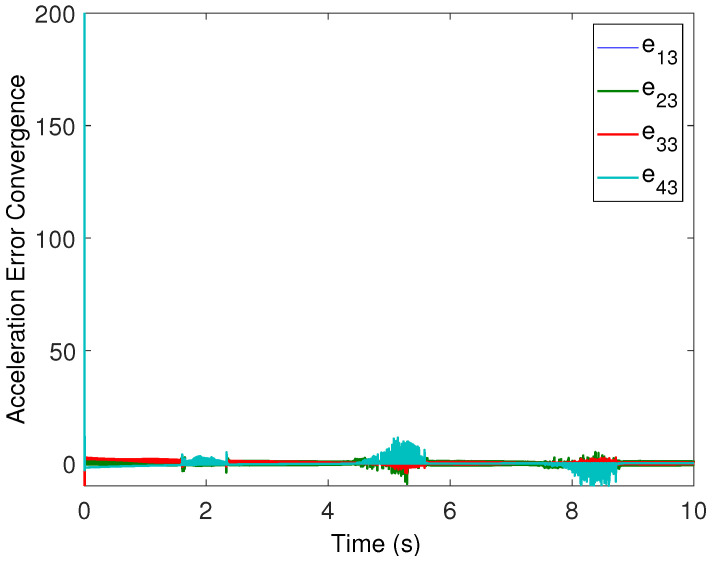
Acceleration errors’ convergence.

All the followers were operated under the influence of time-varying sinusoidal disturbances to show the robustness of the proposed distributed control protocols. The consensus in positions, velocities, and accelerations among the followers and leader is displayed in Figure 2, Figure 3 and Figure 4, respectively. The corresponding position errors convergence, velocities error convergence, and accelerations’ errors convergence are shown in Figure 5, Figure 6 and Figure 7, respectively. It is obvious that the consensus among the states of leader and followers is quite fascinating even in the presence of disturbances.

**Figure 8:**
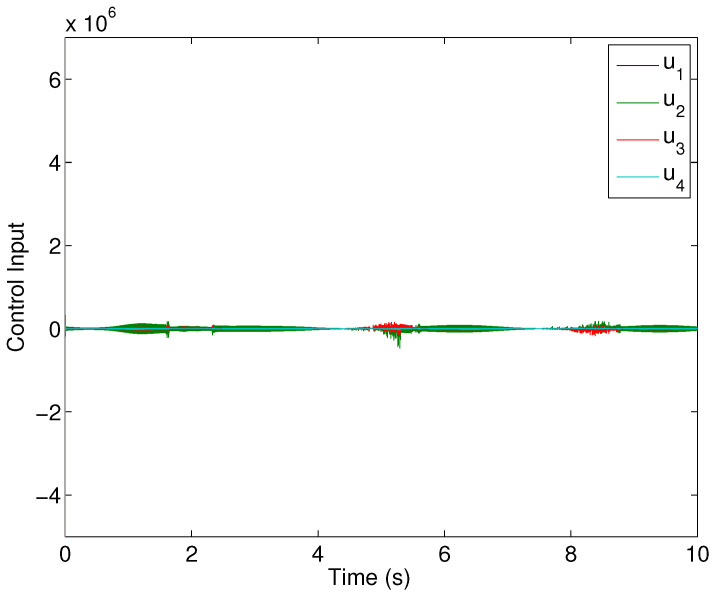
Control inputs’ history.

**Figure 9:**
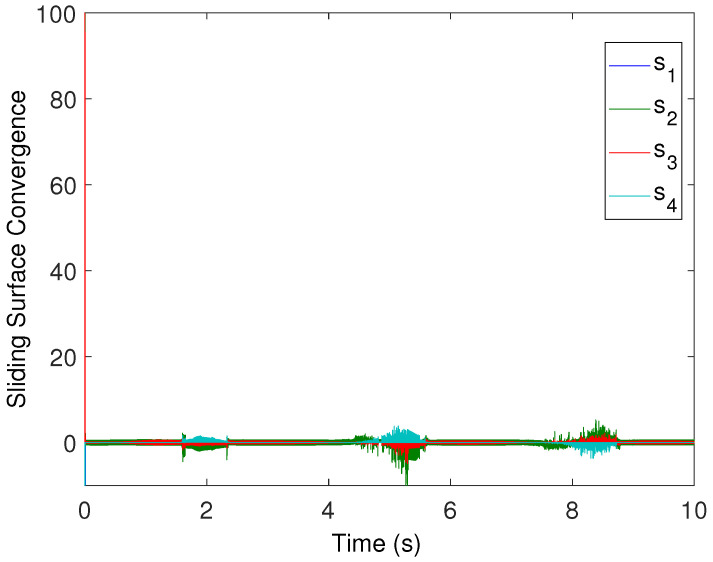
The sliding manifolds convergence from the very start time.

Figure 8 shows the control effort history of each control input. Under the proposed control algorithm, one may obtain almost noise-free control, which reduces chattering (because the noises also cause chattering). The relevant sliding manifolds, which ensure that sliding mode from the very start (as shown in Figure 9) converges to zero in finite time, which ensures the robustness of the designed controller. Having looked at the simulation results, it is evident that the newly designed control protocols offer excellent benefits in terms of robust consensus established from the beginning, which is an appealing attribute of the proposed design. Hence, it is important to conclude that this protocol design is suitable for the consensus of higher-order systems operating under uncertain conditions.

## 6. Conclusions

In this paper, a network of higher-order nonlinear uncertain agents was considered. The network topology was fixed and was based on a directed graph. The main task was to meet consensus among the leader and *n* followers. For this purpose, consensus error dynamics were developed, and a novel sliding surface, analogous to proportional-integral type, was considered. The designed control protocol was capable enough to establish sliding mode along the designed sliding surface from the very beginning. In sliding mode, the error dynamics evolved with full states, which were governed by terminal attractors [27]. This confirmed the finite-time consensus errors convergence to equilibrium. This finite time convergence generally results in high precision. In addition, robustness was enhanced from the very beginning because of the reaching phase elimination. Rigorous mathematical stability analysis is presented, and the simulation results are presented to illustrate the benefits of the newly designed distributed control protocols. The results confirmed that the newly designed law is an interesting candidate for higher-order uncertain systems.

## Figures and Tables

**Table 1:** Parameters of the controllers used in the simulation.

$\alpha_{11}$	$\alpha_{12}$	$\alpha_{13}$	$\alpha_{14}$	$\alpha_{21}$	$\alpha_{22}$	$\alpha_{23}$	$\alpha_{24}$
0.5	0.2	0.02	0.22	0.12	0.32	0.12	0.02
$\alpha_{31}$	$\alpha_{32}$	$\alpha_{33}$	$\alpha_{34}$	$\alpha_{41}$	$\alpha_{42}$	$\alpha_{43}$	$\alpha_{44}$
0.3	0.32	0.42	0.22	0.12	0.12	0.21	0.22
$\beta_{11}$	$\beta_{12}$	$\beta_{13}$	$\beta_{14}$	$\beta_{21}$	$\beta_{22}$	$\beta_{23}$	$\beta_{24}$
0.01	0.22	0.22	0.32	0.22	0.32	0.12	0.42
$\beta_{31}$	$\beta_{32}$	$\beta_{33}$	$\beta_{34}$	$\beta_{41}$	$\beta_{42}$	$\beta_{43}$	$\beta_{44}$
0.5	0.02	0.42	0.22	0.2	0.52	0.42	0.52
$b_{11}$	$b_{12}$	$b_{13}$	$b_{14}$	$b_{21}$	$b_{22}$	$b_{23}$	$b_{24}$
15	21.2	15.2	15.2	25.2	8.2	15.2	6.2
$b_{31}$	$b_{32}$	$b_{33}$	$b_{34}$	$b_{41}$	$b_{42}$	$b_{43}$	$b_{44}$
10	20.2	25.2	81.2	14.2	4.2	25.2	23.2
$c_{11}$	$c_{12}$	$c_{13}$	$c_{14}$	$c_{21}$	$c_{22}$	$c_{23}$	$c_{24}$
5.4	25.2	15.2	35.2	45.2	18.2	15.2	25.2
$c_{31}$	$c_{32}$	$c_{33}$	$c_{34}$	$c_{41}$	$c_{42}$	$c_{43}$	$c_{44}$
5.6	2.2	15.2	5.2	25.2	8.2	22.2	6.2

## Data Availability

Not applicable.
